# Supplementary material for: Cervicovaginal levels of human beta defensins during bacterial vaginosis
Source: PLoS One. 2021 Dec 2;16(12):e0260753. doi: 10.1371/journal.pone.0260753 (PMC8639056; doi:10.1371/journal.pone.0260753)
Supplement: Attachment — Submitted filename: RESPONSE TO REVIEWERS.pdf [file pone.0260753.s002.pdf]

RESPONSE TO REVIEWERS

Journal Requirements:

Please review your reference list to ensure that it is complete and correct. If you have cited papers that have been retracted, please include the rationale for doing so in the manuscript text, or remove these references and replace them with relevant current references. Any changes to the reference list should be mentioned in the rebuttal letter that accompanies your revised manuscript. If you need to cite a retracted article, indicate the article's retracted status in the References list and also include a citation and full reference for the retraction notice.

Answer: We found a typo in the title of reference 45 which was corrected, and some formatting errors along the references which were corrected as well. We did not cite papers retracted.

Comments to the Author

1. If the authors have adequately addressed your comments raised in a previous round of review and you feel that this manuscript is now acceptable for publication, you may indicate that here to bypass the "Comments to the Author" section, enter your conflict of interest statement in the "Confidential to Editor" section, and submit your "Accept" recommendation.

Reviewer #2: (No Response)

Reviewer #3: (No Response)

---

2. Is the manuscript technically sound, and do the data support the conclusions?

The manuscript must describe a technically sound piece of scientific research with data that supports the conclusions. Experiments must have been conducted rigorously, with appropriate controls, replication, and sample sizes. The conclusions must be drawn appropriately based on the data presented.

Reviewer #2: Partly

Reviewer #3: Yes

---

3. Has the statistical analysis been performed appropriately and rigorously?

Reviewer #2: Yes

Reviewer #3: I Don't Know

---

4. Have the authors made all data underlying the findings in their manuscript fully available?

The PLOS Data policy requires authors to make all data underlying the findings described in their manuscript fully available without restriction, with rare exception (please refer to the Data Availability Statement in the manuscript PDF file). The data should be provided as part of the manuscript or its supporting information, or deposited to a public repository. For example, in addition to summary statistics, the data points behind means, medians and variance measures should be available. If there are restrictions on publicly sharing data—e.g. participant privacy or use of data from a third party—those must be specified.

Reviewer #2: Yes

Reviewer #3: Yes

---

5. Is the manuscript presented in an intelligible fashion and written in standard English?

PLOS ONE does not copyedit accepted manuscripts, so the language in submitted articles must be clear, correct, and unambiguous. Any typographical or grammatical errors should be corrected at revision, so please note any specific errors here.

Reviewer #2: Yes

Reviewer #3: Yes

---

6. Review Comments to the Author

Please use the space provided to explain your answers to the questions above. You may also include additional comments for the author, including concerns about dual publication, research ethics, or publication ethics. (Please upload your review as an attachment if it exceeds 20,000 characters)

Reviewer #2: The manuscript is considerably improved and the authors have addressed many of the editorial comments. There are still some careless inconsistencies throughout and these should be addressed with thought for the overall flow of the discussion.

A major strength of the study lies in the number of individuals which have been

included in the analysis, but the interpretation of the associations seen is hampered by the limited analysis performed so the authors should be careful not to over interpret their findings.

Specifically, the authors should caution against drawing firm conclusions about the association Lactobacilli spp and levels of hBD as they have not characterised the bacterial composition of the swabs. Statements such as line 224 “ Thus, the production presence of hBDs is related to the presence of a increased abundance of Lactobacillus spp. in the vaginal microbiota “

should be modified accordingly to be more consistent with

line 283 “Thus, we postulate may hypothesize that the decreased lower levels of hBD-1, -2 and -3 in the presence of BV could be, in part, due to the production of proteases by the complex highly diverse bacterial community of BV,”

Answer: We thank the reviewer for this pointing this out. Then, we removed this sentence “ Thus, the production presence of hBDs is related to the presence of a increased abundance of Lactobacillus spp. in the vaginal microbiota “.

Importantly, a multivariate analysis has been carried out to explore the nature of the associations observed in the univariate analysis. The levels of HBD 1-3 remain significantly associated with BV as do the number of sexual partners and use of hormonal contraception. The authors discuss their findings in the context of previous data, and perhaps could suggest additional future analyses in their discussion which might provide mechanistic insights into the associations that they describe (line 289)

Answer: We do agree with the reviewer that this information is relevant and should be included in the text. We just added this information at the discuss section in last paragraph.

There remain some careless “typos”;
Line 172 The concentrations of hBD are still 1000X too low. These errors have been corrected elsewhere in the manuscript and this is a careless oversight.

Answer: In fact we have detected some typos errors and have rewritten according.

Reviewer #3: The manuscript "Cervicovaginal levels of human beta defensins during bacterial vaginosis" addresses a complex and controversial issue that has been under research thoroughly for the last years: the relationship between vaginal microbiota and endogenous inflammatory factors and regulation.

I agreed with most of the comments and observations of the two previous

reviewers and found that the authors have addressed them appropriately. After the corrections and additions made, the manuscript has significantly improved.

Even when the methodology used is simple and does not allow a more profound analysis (such as characterization of Lactobacillus species), the conclusions are valuable and contribute to this field's knowledge.

Thus, it's my recommendation to accept it for publication.

---

7. PLOS authors have the option to publish the peer review history of their article (what does this mean?). If published, this will include your full peer review and any attached files.

If you choose “no”, your identity will remain anonymous but your review may still be made public.

Do you want your identity to be public for this peer review? For information about this choice, including consent withdrawal, please see our Privacy Policy.

Reviewer #2: No

Reviewer #3: No
